# PromoACTIVA-SC: A Tool Aiming at Identifying Health Promotion Practice of Civil Society Organizations

**DOI:** 10.3390/healthcare13172097

**Published:** 2025-08-23

**Authors:** Olga Lopez-Dicastillo, Andrea Iriarte-Roteta, Elena Antoñanzas-Baztán, Sara Sola-Cia, Agurtzane Mujika, Naia Hernantes, Isabel Antón-Solanas, María Anunciación Jiménez-Marcos, Edurne Zabaleta-del-Olmo, Dolors Juvinyà-Canal, María Jesús Pumar-Méndez

**Affiliations:** 1 Department of Health Sciences, Universidad Pública de Navarra-UPNA, 31008 Pamplona, Spain; olga.lopezdicastillo@unavarra.es (O.L.-D.); elena.antonanzas@unavarra.es (E.A.-B.); sara.sola@unavarra.es (S.S.-C.); anunciacion.jimenez@unavarra.es (M.A.J.-M.); mj.pumarmendez@unavarra.es (M.J.P.-M.); 2 IdiSNA, Navarra Institute for Health Research, 31008 Pamplona, Spain; 3 CreaP Research Group, Universidad Pública de Navarra-UPNA, 31008 Pamplona, Spain; 4 Navarra Health Care Service, 31008 Pamplona, Spain; 5 Department of Health, Government of Navarra, 31008 Pamplona, Spain; 6 SILO Research Group, Nursing II Department, Faculty of Medicine and Nursing, UPV/EHU, Paseo Dr. Beguiristain 105, 20014 Donostia, Spain; agurtzane.mujika@ehu.eus (A.M.); naia.hernantes@ehu.eus (N.H.); 7 Department of Physiatry and Nursing, Faculty of Health Sciences, University of Zaragoza, C/Domingo Miral s/n, 50009 Zaragoza, Spain; ianton@unizar.es; 8 Research Group SAPIENF (B53_23R), University of Zaragoza, 50009 Zaragoza, Spain; 9 Fundació Institut Universitari per a la Recerca a l’Atenció Primària de Salut Jordi Gol i Gurina (IDIAPJGol), 08007 Barcelona, Spain; ezabaleta@idiapjgol.org; 10 Gerència d’Atenció Primària Barcelona Ciutat, Institut Català de la Salut, 8007 Barcelona, Spain; 11 Departament d’Infermería, Universitat de Girona, 17004 Girona, Spain; dolors.juvinya@udg.edu; 12 Grup de Recerca Salut i Atenció Sanitària, Universitat de Girona, 17004 Girona, Spain; 13 Càtedra de Promoció de la Salut, Universitat de Girona, 17004 Girona, Spain

**Keywords:** capacity building, civil society organizations, health promotion, questionnaire design

## Abstract

**Background:** The World Health Organization (WHO) defines health promotion as the process of enabling individuals to gain control over and improve their health. This shifts the focus from lifestyle choices to broader social determinants of health, requiring involvement from healthcare, authorities, industry, civil society, and the media. Civil society engagement in health initiatives offers benefits such as empowerment, service delivery, flexibility, policy participation, and credibility. However, identifying specific health promotion actions for civil society organizations (CSOs) is challenging. The lack of assessment tools for CSOs hinders evaluation and improvement. **Objective**: To develop a tool, ‘PromoACTIVA-SC’, to assess CSOs’ health promotion practice by identifying essential actions that constitute the health promotion process. **Methods**: ‘PromoACTIVA-SC’ was developed through documentary analysis and validated by experts. CSOs’ members participated in cognitive interviews for comprehensibility, and the tool was pilot tested for administration and Likert scale evaluation. **Results**: The final questionnaire, consisting of 8 phases and 40 items, demonstrated good content validity. Its use can help to map CSOs’ practices and identify areas needing improvement. CSOs can use it for self-assessment and in collaborative health promotion and disease prevention efforts. **Conclusions**: ‘PromoACTIVA-SC’ is the first tool designed to assess civil society’s role in the health promotion process. Its future use will reveal the extent to which civil society organizations actively participate in health promotion. It can also be used to promote CSOs’ involvement in health policy and administration, enhancing public health outcomes through collaborative, cross-sectoral efforts.

## 1. Introduction

The World Health Organization (WHO) defined health as ‘a state of complete physical, mental, and social well-being and not merely the absence of disease or infirmity’ [1]. Although the word ‘complete’ might make the concept difficult to achieve, it was included as a term to show a holistic approach to health rather than a perfect one [2]. The Ottawa Charter added that health is ‘a resource for everyday life, not the objective of living. Health is a positive concept emphasizing social and personal resources, as well as physical capacities’ [1]. Thus, health is a multifarious instrument, including external and internal resources, for a good life [2].

Health promotion is a social and political process. It encompasses actions aimed at strengthening individuals’ skills and capacities and changing social, environmental, and economic conditions to mitigate health impacts [1]. It requires the involvement of multiple sectors including health, local authorities, industry, civil society, and the media [1].

All sectors involved need to function independently and collaboratively to address health and well-being holistically. Effective health promotion requires coordinated intersectoral action to improve health or influence its determinants [3]. Intersectoral collaboration requires that a ‘group of autonomous stakeholders of a problem domain engage in an interactive process, using shared rules, norms and structures, to act or decide on issues related to that domain’ [4].

Focusing on the procedural nature of intersectoral collaboration may be instrumental in overcoming one of its most frequently cited barriers, namely the lack of a common language for health [5,6]. Viewing health promotion as a process rather than an outcome entails understanding it as “a series of actions which are carried out in order to achieve a particular result” [7]. Adopting this lens can help create a common approach for intersectoral collaboration by providing all stakeholders with a shared structure and vocabulary. When health promotion is broken down into agreed stages, such as planning, implementation, and evaluation, and the actions within each stage are explicitly defined, stakeholders from different sectors can align their terminology to describe their respective tasks and responsibilities. This allows for a collective understanding of the sequence and purpose of health promotion activities, making communication more precise and needs for coordination more concrete, while reducing misunderstandings arising from sector-specific jargon. This process-oriented perspective on health promotion is still novel, and existing literature offers limited guidance for different stakeholders. Most studies have focused on describing particular health promotion interventions in isolation rather than considering the entire process in which those interventions are situated. An exception is the work of Pumar-Méndez et al. [8], which analyzed health promotion as a process in a specific sector, primary healthcare. The authors identified eight action areas: planning; situational analysis; capacity building; awareness and public opinion development; advocacy; network development; alliance building; and intervention strategies. Each phase includes a set of actions to be undertaken by health professionals. To the best of the authors’ knowledge, similar detailed process-oriented guidelines have only recently been designed for sectors such as local governments [9], educational settings [10], and public health [11]. Although they have been implemented in a research project, they have not yet been applied extensively [12]. Their full development and testing, as well as the creation of new tools for other key sectors, would be of great value for stakeholders seeking to adopt a process perspective and thereby facilitate intersectoral collaboration.

Civil society is one of these stakeholders and should be considered a priority, as it is a key actor in the health promotion process and an essential partner in fostering intersectoral collaboration. Working with society has become a key strategy for the WHO European Region since in 2012 the WHO adopted the whole-of-society approach as part of the Health 2020 framework. This emphasizes the importance of engaging civil society, communities, and individuals to reinforce community resilience against health, security, and well-being threats. It also complements the whole-of-government approach, which focuses on cross-sector government collaboration to achieve a common goal [13]. However, complementation of the aforementioned approaches often faces challenges, including lack of or poor civil society representation in contexts where decisions impacting the social determinants of health are made; governance barriers for genuine civil society participation; and financial constraints. In addition, tensions between the public sector and civil society with administrative procedures are commonly disharmonized, complicating further successful partnerships [14].

Defining civil society poses a challenge, as the terms ‘Civil society’ or ‘society’ are either comprehensive or general terms that encompass diverse people and organizations. The use of the term ‘civil society organizations’ (CSOs) as opposed to ‘civil society’ helps characterize the civil society of a country and analyze their roles and interactions [14]. To provide analytical clarity, this study adopts Anheier’s operational definition of CSOs [15] as the sphere between family, state, and market, where individuals voluntarily associate to advance shared interests. CSOs are thus understood as institutions and groups characterized by three key criteria: (1) voluntary participation, (2) autonomy from state, market, and family structures, and (3) collective action to advance common interests. In this study, particular focus will be placed on the following CSOs: voluntary associations, non-governmental organizations (NGO), nonprofits, foundations, charities, social movements, civic groups, networks, and coalitions.

Engaging civil society in health has been proven beneficial to their empowerment, flexibility, responsiveness, government commitment, policy participation, and increased credibility [14]. In addition, CSOs address issues that neither the state, nor the market or the families would be concerned with or would be able to address rapidly [14]. Despite all these benefits, CSOs often manage health-related aspects in isolation or assume responsibilities that other sectors should also share. Their health-related activities include one or more of the following three: policy, service provision, and governance (see Table 1).

While these categories capture the main types of health-related activities undertaken by CSOs, the literature does not usually specify them in relation to health promotion nor situate them within a process perspective. As explained above, making this translation could support CSOs in adopting a process-oriented perspective on health promotion, providing them with the shared structure and vocabulary needed to overcome their isolation and role overlap with other sectors. This, in turn, can strengthen CSOs’ health promotion practice and their contribution to intersectoral collaboration.

Building on this rationale, the objective of this study is to design and test a tool that identifies the essential actions of health promotion practice within CSOs, and that enables them to assess their current practice in relation to this process.

## 2. Materials and Methods

**Figure 1:**
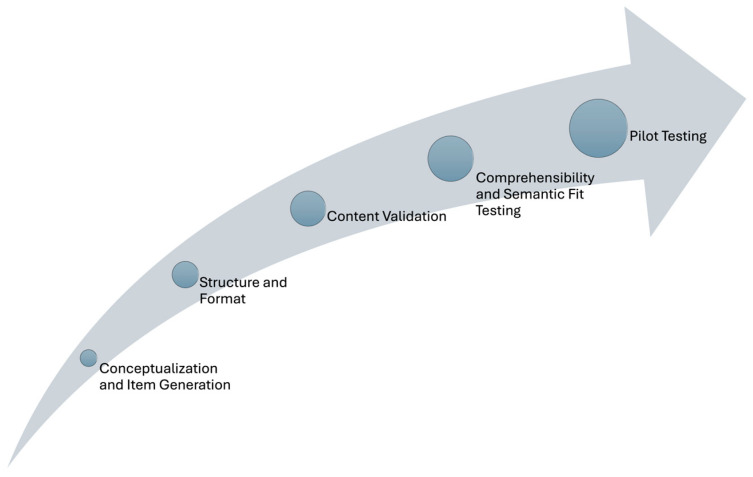
Tool development stages.

PromoACTIVA-SC was conceptualized understanding health promotion practice as a formative construct, where the items capture distinct dimensions of CSOs engagement in health promotion practice rather than serving as interchangeable indicators of a single latent variable [16]. The design and validation of PromoACTIVA-SC followed these stages: conceptualization and item generation; structural, format, and content validity analysis; comprehensibility, semantic fit, and pilot testing (see Figure 1). The stages of content validity, comprehensibility, and feasibility were of special importance, rather than focusing on internal consistency, since the items were not expected to be correlated in formative construct tools.

### 2.1. Conceptualization and Item Generation

A documentary analysis was conducted to compile the set of actions CSO members must perform for a comprehensive health promotion practice. Five literature searches were conducted in PubMed using the Ottawa Charter action areas as the theoretical framework. PubMed was selected for its broad coverage of peer-reviewed biomedical and public health literature, and the Charter domains were chosen to ensure alignment with internationally recognized health promotion principles. Search terms were derived directly from these action areas and iteratively refined through preliminary searches and team discussions to balance sensitivity (capturing a wide range of potentially relevant documents) with specificity (excluding unrelated material). The final strategies for each search are detailed in Table S1. To ensure the analysis reflected recent and accessible evidence, the searches were limited to publications in Spanish and English from 2011 to 2021. Gray literature was not included to prioritize methodological rigor and reproducibility, as peer-reviewed publications provide greater consistency in reporting and allow clearer comparison across studies. Purposeful sampling [17] was used to select the documents based on the following inclusion criteria:

1. They should address civil society organizations, understanding them as voluntary associations, NGOs, nonprofits, foundations, charities, social movements, civic action groups, networks, and coalitions.
2. They had to describe either actions or omissions regarding health promotion actions performed by CSOs.

Documents that focused solely on prevention or referred to CSOs’ immediate family, market, or state were excluded. Snowballing was used to identify additional relevant documents by reviewing references of initially selected documents.

Outputs were reviewed and analyzed using a thematic framework analysis [18]. The framework used was the Ottawa Charter actions as defined and detailed by Fry and Zask [19]. The analysis was conducted in three steps: the familiarization with the data usage; the codification of the data (including document coding and creating a list of codes aligned to the five actions proposed by the Ottawa Charter); and the interpretation and grouping of the codes into categories based on the type of action. Three researchers took part in this analysis, repeatedly reading the documents, cross-checking their coding, and reviewing duplications and disagreements regarding codes and categories.

For the final interpretation step, the codes were rearranged according to health promotion phases as described by Pumar-Mendez et al. [8] in their taxonomy for primary healthcare. Definitions were then provided for the main categories of the process.

Once the classification was completed, the items were reviewed for completeness and relevance to the national context and for intersectoral collaboration. Regular team meetings ensured a rigorous, iterative, and consensus-driven analysis.

### 2.2. Structure and Format

After finalizing the items, they were translated into Spanish and reviewed using De Vaus’ [20] checklist of 17 questions to ensure appropriate editing, clarity, and to avoid ambiguity.

A 5-point Likert-type scale was selected to assess respondents’ perceptions of the degree of execution of each action by their CSO: 1 = none (the action is not performed), 2 = insufficient (the action is being considered for implementation), 3 = sufficient (the action is planned and has been partially initiated), 4 = advanced (the action is carried out but not yet systematized), and 5 = very advanced (the action is systematically carried out and integrated into the organization’s practice).

Attention was given to the format of PromoACTIVA-SC to ensure that participants could easily understand and respond. Literature recommendations were followed to include a section outlining the tool’s aim, instructions for completion and submission, criteria for participant selection, confidentiality aspects, and an explanation about its structure [21].

### 2.3. Content Validation

To evaluate the content of PromoACTIVA-SC, a panel of experts was formed, comprising 8 to 12 experts as recommended by the literature [22]. Experts in this study were defined as professionals knowledgeable in health promotion concepts and either belonged to CSOs or were well-acquainted with them from a theoretical or academic perspective. Twelve experts were contacted by phone, informed about the project, and then sent PromoACTIVA-SC and the form via email.

The form was designed according to Polit et al.’s recommendations [22]. Experts were asked to analyze PromoACTIVA-SC’s items for relevance and clarity using a four-point Likert-type scale (with 4 being the highest and 1 the lowest). They could add observations regarding every item, suggest new items, and provide general feedback [23]. Additionally, their sociodemographic characteristics were collected.

Upon receiving the completed forms, the content validity index for each item (I-CVI) was calculated to guide decisions on item revision or rejection. The I-CVI was determined by the ratio of experts who rated an item 3 or 4 to the total number of experts who participated [22].

Items scoring 0.78 or higher were deemed excellent. Items scoring below 0.78 were reviewed based on expert suggestions, and those scoring below 0.6 were considered for elimination [22]. New items were included if 80% of the experts recommended them.

The overall validity of PromoACTIVA-SC (SCVI/ave) was calculated as the ratio of the sum of all I-CVIs to the total number of items. A score of 0.90 or higher was considered indicative of excellent content validity [22].

### 2.4. Comprehensibility and Semantic Fit Testing

Volunteers, members, or employees who led local CSOs or held management roles were invited to participate in cognitive interviews. This stage aimed at assessing how they understood, mentally processed, and responded to the items [24]. Participants were purposively selected from a variety of CSOs involved in diverse health-related areas (breastfeeding, addictions, communicable and non-communicable diseases, and ethnic minorities), and with different roles in the organization. This helped test PROMOACTIVA-SC with a diverse group of participants representing various health promotion goals and social groups.

Participants at this stage were contacted by phone and email, provided with verbal and written information about the project, and signed a consent form.

Two researchers conducted each interview: one leading the interview and the other providing organizational and technical support. Interviews were audio-recorded, and responses were documented separately by both researchers. To minimize variability among the four experienced researchers involved, a standardized guide was developed, incorporating the think-aloud technique and probing questions (see guide in Table 2). A form was designed to collect data on the interview process, item interpretation, and the time taken by participants to start explaining each item’s meaning. An interpretation time exceeding 5 s was considered indicative of comprehension difficulties [24]. Specific problems or suggested changes in the items noted by the participants were also recorded [25].

After the interviews, the forms were analyzed, and the researchers compared their notes and re-listened to the recordings. All four researchers convened to review the reasons behind any incorrect item interpretations, listening to the recorded interviews for additional context.

Special attention was given to instances where participants might have misunderstood certain concepts, using them differently than intended, or were distracted while reading or explaining aloud the items. Decisions about item revisions were made when two or more participants misinterpreted an item, made a recommendation, or suggested a change.

### 2.5. Pilot Testing

Pilot testing evaluated PromoACTIVA-SC’s administration process and its ability to differentiate extreme responses [26]. As in the previous stage, purposive sampling was used to recruit participants from CSOs working in diverse health-related fields and occupying different roles within their organizations. This strategy ensured continuity in capturing a broad spectrum of perspectives while also allowing us to test PromoACTIVA-SC across varied health promotion contexts and organizational settings.

To examine floor and ceiling effects, a pilot study with at least 30 participants similar to the final target audience is necessary [27,28,29]. PromoACTIVA-SC was distributed in paper format, and participants were given two weeks to complete it, with a reminder sent after one week.

A descriptive analysis of sociodemographic variables was conducted, along with a frequency analysis of each item. Missing data were also examined. Items showing a ceiling effect (defined as more than 70% responses in the two highest options) would prompt a review of the Likert scale [30].

### 2.6. Ethical Considerations

Regarding ethical considerations, in addition to obtaining ethical approval from the relevant university committee and informed consent from participants, all stages of the study adhered to ethical research principles. This included safeguarding data privacy, ensuring that participation was voluntary, and emphasizing participants’ right to withdraw at any point without consequence. All data were anonymized and stored securely, accessible only to the research team.

## 3. Results

PromoACTIVA-SC is the tool developed, comprising 40 items grouped into 8 phases that assess health promotion actions that CSOs need to perform to fully implement health promotion as a process. Each action is measured using a 5-point Likert scale. The results are presented in the following sections according to the design and validation procedures.

### 3.1. Conceptualization and Item Generation

**Figure 2:**
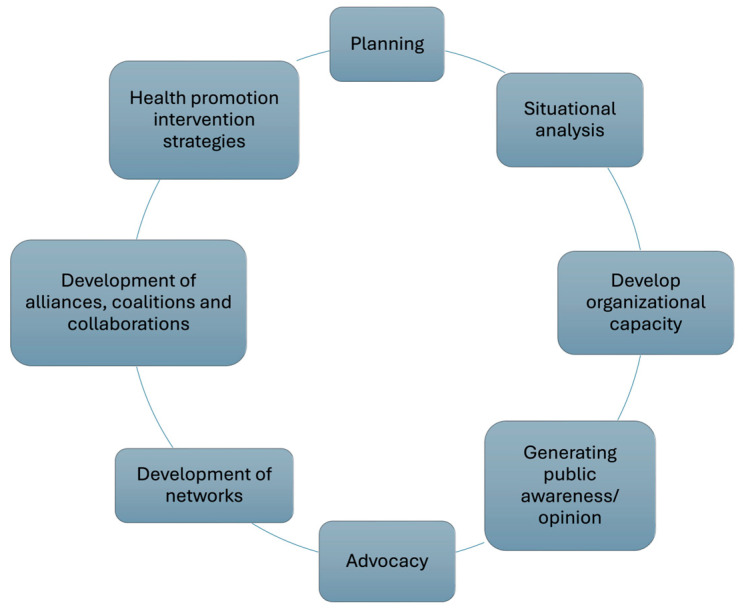
Phases of the health promotion process.

A total of 60 actions were identified from the revised documents (see Table S1 for searches and outcomes) and drafted into item form (see Table S2). These items were then reviewed and categorized into the 8 phases of the health promotion process (see Figure 2) as identified by Pumar-Méndez et al. [12]. This analysis led to the merging of some items, considering them transversal or part of others. After applying De Vaus’ [20] criteria, the total number of items was reduced to 36 (see Table S3). Four additional items (see items in Table S3) were included after reviewing the entire instrument for completeness and relevance to the national context. Consequently, a 40-item questionnaire was developed, along with 15 sociodemographic questions (7 about the CSO and 8 about the participant).

### 3.2. Structure and Format

PromoACTIVA-SC consisted of 8 A4 pages. The first page included the presentation of the tool’s aim, with instructions on how to complete and return it, participant selection criteria, and confidentiality aspects including an explanation about its structure. The second and third pages contained 15 sociodemographic questions about the participants’ CSOs. Attention was given to the overall presentation of PromoACTIVA-SC to ensure it was user-friendly and clearly formatted.

### 3.3. Content Validation

Ten out of the twelve experts invited to participate in a panel returned the form (see Table 3 for expert characteristics, professions, and areas of expertise). The overall presentation of PromoACTIVA-SC was deemed clear and well-valued by the experts, although three noted its length. The content validity was excellent (S-CVI/Ave = 0.9025). All the items except one received I-CVI scores between 0.80 and 1 (16 items had I-CVI = 1, 13 items had I-CVI = 0.90, and 10 items had I-CVI = 0.80) (see Table S3).

Item 25, “Advocating for the reorientation of the health sector,” received a low I-CVI score (0.40). However, the qualitative feedback suggested that expert concerns were not about the irrelevance of the concept but rather about overlap and wording. Specifically, two experts noted similarity with Item 24 (Advocating for changes in healthy public policies for the development of safe and healthy environments), three found the phrasing too general or ambiguous, and one questioned the utility of reorienting health services. To address these concerns, the item was revised for greater precision as ‘Advocating for the reorientation of the health sector towards health promotion.’ Given the central role of health sector reorientation in the literature and the strategic importance of CSOs in advancing this agenda, the item was considered conceptually essential. Therefore, it was retained, while acknowledging the need to further test its clarity and comprehensibility in the next phase.

Regarding clarity, 20 items were reviewed based on expert recommendations (see Table 4 for decisions made). Experts identified four items that measured multiple aspects and recommended separating them. One item required term clarification, due to potential respondent confusion. Five items needed more precision, and four items were rewritten. Two items underwent minor changes for language consistency. Three items were retained for cognitive interviews as experts suggested changing them, considering that respondents would not understand them. One redundant item was eliminated upon experts’ agreement. These revisions resulted in a new version of PromoACTIVA-SC, now consisting of 41 items.

The Likert scale used to measure the items was deemed adequate, although one expert suggested including an option for respondents to indicate if they did not know the answer. Consequently, an option ‘D’ (Desconozco, in English, ‘I don’t know it’) was included to allow respondents to indicate when they were unsure if an action was executed in their organization.

Sociodemographic questions were slightly modified following recommendations. The final set included 12 questions about the CSO (five additional questions: one regarding the availability of a CSO’s website; three about the organization’s action area; and one about its funding) and 10 about the participants (two additional questions about the time dedicated to the CSO and the year that the individual started collaborating with CSOs).

### 3.4. Comprehensibility and Semantic Fit Testing

Five out of six participants accepted to participate in the cognitive interviews, of which four were female and one was male. They represented diverse CSOs with different characteristics and addressed topics such as breastfeeding, addictions, communicable and non-communicable diseases, and ethnic minorities. Participants were selected for their deep familiarity with their respective CSOs and active roles in organizational management, including two volunteers, one member, and two employees.

The interviews lasted between 1 and 2 h and revealed that participants took more time to respond to the initial 8 items under the ‘Planification’ category, but generally had no difficulties explaining the items.

As a result, PromoACTIVA-SC was refined to include 40 items. Changes in the items are summarized in the third column of Table 4. During the interview, four participants identified two items as redundant (item 26 and 27 Table S3), leading to the merger of ‘Advocating for increasing infrastructure for health promotion’ and ‘Advocating for increasing resources for health promotion’ into the latter (item 27 in Table S3), as participants considered the ‘infrastructure’ a ‘resource’.

Item 8 (Table S3) was revised to clarify the definition of ‘Community health diagnoses’ as it was deemed challenging for respondents (three participants did not understand the term). The item was rewritten as: ‘Elaborating the community health diagnosis (Use and collection of sociodemographic, education, health geographical, sociocultural, problems, needs, and resources)’.

Furthermore, three participants had difficulty understanding the item ‘36 Participating in the community health council’. An explanatory note was added to clarify, now formulated as ’36 Participating in the community health council (citizen participation and representation body)’.

To enhance usability, the general structure of PromoACTIVA-SC was slightly adjusted into three parts, as participants were distracted by the collection of sociodemographic data from recalling the Likert scale explanation, which was essential to later respond to the items. Therefore, the first part included a presentation, detailing its intended audience, objectives, and structure.

The second part featured 21 questions on sociodemographic characteristics, with questions about CSOs reduced to 11 (consolidating two questions on organization’s action area and including ‘Is your organization part of any national or international platform or federation?’ as worded by participants). The 10 questions on participant sociodemographic characteristics remained unchanged.

Lastly, the third part provided an initial description of the 8 phases and the 40 items next in line, alongside the explanation of the Likert scale and guidance on completing PromoACTIVA-SC.

Explicit clarifications were added in the definition of each phase to specify that the items referred to the organization and not to participants themselves, addressing confusion noted by two participants.

### 3.5. Pilot Testing

PromoACTIVA-SC was distributed to 35 participants with different roles within 12 different CSOs, with 31 participants returning the questionnaire (which translated to 88.6% response rate). A summary of their characteristics is in Table 5.

The response percentages according to the scale values are provided in Table S4 in the Supplementary Material. Most of the items (37) showed dispersed response patterns. Missing data were minimal (3.2%) in 12 items. Notably, only three items exhibited a ceiling effect (items 18. ‘Developing materials and tools for the practice of health promotion’, 32. ‘Referring persons to services available in the community’ (and 39. ‘Developing health promotion programs’). These three items showed 77.53%, 77.4%, and 70.9% of responses in the two highest options, respectively; however, they represent actions commonly undertaken by CSOs, therefore deeming the Likert Scale optimal and retaining it as proposed.

During the data collection phase, it was discovered that there were no active community health councils in the region. Nevertheless, 48.4% of the respondents reported varying degrees of engagement with Item 36. This led to follow-up emails to clarify their rationale and responses. It emerged that some CSOs actively participated in community councils, commissions, and forums that, while not exclusively health-related, addressed critical factors such as housing, environment, or immigration. This finding led to changing the item in the final version of PromoACTIVA-SC, reflecting better what the situation of CSOs was. The item was changed to ‘Participating in representation and citizen participation bodies in which aspects that influence health are dealt with (e.g., Health Councils, Commissions and Forums)’.

The final version of PromoACTIVA-SC can be found as Supplementary Material (S1).

## 4. Discussion

PromoACTIVA-SC is a tool designed to assess the comprehensive implementation of the health promotion process within CSOs. It comprises 40 items grouped into 8 phases that reflect the health promotion process and collects 21 variables from participants and the CSO they subscribe to. It was designed rigorously through a documentary analysis to identify conceptual aspects for inclusion and item formulation. The validation process included content validity analysis, comprehensibility and semantic fit testing, using expert validation and cognitive interviews. Within a broader project [12], empirical data that may support further refinement have already been collected but are still under analysis and will be reported in future publications. However, publishing the tool at this stage is pivotal, as it sets its use in motion and enables early validation and adaptation across different contexts.

PromoACTIVA-SC serves as an evaluation or guiding tool for CSOs aiming at implementing health promotion processes that contribute to a wide range of health topics across the domains of well-being, including physical, mental, and social health. It can therefore support CSOs’ contributions to a variety of areas, including timely and highly relevant ones such as physical activity promotion. PromoACTIVA-SC can also promote capacity building within organizations, elucidating CSOs’ roles and responsibilities in health promotion [31], and approaching often theoretical action areas more practically. This tool transcends mere lifestyle promotion or health education, recognizing CSOs’ pivotal involvement in policy advocacy and governance for health promotion [14].

The utility of PromoACTIVA-SC extends beyond internal evaluation, as it provides insights for local governments to better allocate resources and funding to CSOs. Furthermore, CSOs can leverage this information to elaborate investment proposals or attract external funders or volunteers.

The insights gleaned from PromoACTIVA-SC will be essential to enhancing health promotion practices within CSOs and fostering improved collaboration across sectors. Intersectoral collaboration could be strengthened with the development of similar tools tailored for primary healthcare, schools, local governments, and public health entities [12]. In the future, the creation of analogous tools in other sectors would establish a shared language to effectively address key health promotion challenges and explore potential solutions [32].

Standardizing language and phrases across sectors can help enhance collaborative efforts. Evidence shows that actions confined to a single sector often yield limited impact and reach, with interventions potentially lacking effectiveness, efficiency, or long-term sustainability [33]. This collaborative approach may prove advantageous when promoting health at the local level, facilitating cross-sectoral cooperation to identify primary goals and strategies that respond to local priorities and needs, thereby improving the population’s health [34]. Furthermore, this approach is crucial on a global scale to contribute effectively to the Sustainable Development Goals (SDGs), given the interconnected nature of human health, well-being, and sustainable development [33]. Strengthening CSOs’ intersectoral collaboration may be particularly valuable for advancing (health and well-being), SDG 10 (reducing inequalities), and SDG 17 (partnerships), since these goals are rooted in coordinated, cross-sectoral approaches that no single sector can achieve alone. CSOs have a recognized capacity to act as watchdogs and trusted intermediaries, often perceived as free from conflicts of interest, and therefore uniquely positioned to bridge communities, governments, and diverse sectors in pursuit of shared sustainable development objectives. PromoACTIVA-SC can further support this role of CSOs by providing a structured process to help them reflect on how actions in each phase could contribute to the SDGs. The documentary analysis conducted facilitated the identification of core concepts and items incorporated into PromoACTIVA-SC. Specifically, the review focused exclusively on health scientific literature to ensure rigor and relevance, excluding less formal aspects and non-health-related literature. However, expert content validation confirmed the tool’s excellence, highlighting that, although some aspects may not have been fully addressed, important actions were not neglected or ignored.

Some participants perceived PromoACTIVA as lengthy. Nevertheless, its comprehensive nature is essential for thoroughly assessing the health promotion process and obtaining detailed information from CSOs. The identification of specific actions in each phase will facilitate the detection of both actions and omissions, thereby enhancing the implementation of health promotion in the future.

During the research process, a minimum of two researchers participated in each stage. Special attention was devoted to the analysis of the cognitive interviews, with four researchers collaborating to review the data and make informed decisions regarding the items and their content, thus contributing to their reliability [35]. Each stage built upon the previous one, focusing not only on content of PromoACTIVA-SC but also on testing its design, presentation, format, and the Likert scale utilized. This iterative approach likely contributed to its acceptability, evidenced by the high response rate in the pilot study despite its length. This suggests that participants found the tool both meaningful and relevant for their health promotion practice within CSOs. The participants in the pilot testing stage in this study were skewed toward paid employees (71%) and university-educated participants (80,6%), which may limit the generalizability of the findings at this stage. The perspectives of unpaid or less educated groups may be underrepresented and can be explored in the future.

Future research could explore item redundancy or develop a shorter version that improves its usability. However, it is important to recognize that PromoACTIVA-SC is based on a formative construct, that is, its items describe different facets of the construct. When examining the properties of such tools, formative models are required for their analysis. These models assume that the items are not necessarily correlated. Therefore, measurement instruments of this nature do not need to assess metric properties such as the structural validity or internal consistency of scores [36,37]. It is recommended to test the properties of the instrument with larger samples to continue with the validation process suitable for formative models, including convergent validity, indicator collinearity, and statistical significance and relevance of the indicator weights [38].

Another consideration regarding PromoACTIVA-SC’s usability is its novelty. PromoACTIVA-SC offers the first structured framework for CSOs seeking to adopt a process-oriented approach to health promotion, an area where clear guidance has often been lacking. Organizations accustomed to more fragmented practices may find their immediate adoption challenging, as it requires a shift toward more integrated and systematic ways of working. To facilitate adoption, organizations could benefit from the gradual implementation of PromoACTIVA-SC, complemented by reflective exercises and feedback mechanisms that help connect everyday practice with the activities described in its items and phases. Future research could also evaluate strategies for embedding the process-oriented approach to health promotion within CSOs’ organizational culture, building on the foundations provided by PromoACTIVA-SC.

This tool was designed for CSOs as defined in the introduction. This study has excluded some CSOs such as unions, trade associations, and professional or Royal Colleges, which can play a significant role in health-related discussions in many countries. These actors often have distinctive structures, mandates, and ways of engaging in health promotion. For this reason, future studies should consider further refinement and testing of the tool for use in these CSOs and in other types of organizations not included in the present study. This should involve revising the tool’s items in light of the literature on such organizations and piloting and validating it with them before broader application. The methodological process followed in this study can serve as a model for such adaptation. These steps would allow the tool to better reflect their specific roles and practices, thereby enhancing its applicability across diverse organizational settings.

In addition, the tool should undergo a process of cross-cultural adaptation to ensure its conceptual, semantic, and operational equivalence across diverse settings. Although the documentary analysis underpinning the item development was based on international literature, the tool’s initial formulation was in Spanish, and its validation was conducted in the Spanish context. Extending its applicability, therefore, requires not only linguistic translation but also systematic cultural adaptation that takes into account the specific roles, structures, and practices of CSOs in different countries and institutional environments. Indeed, the challenge of using PromoACTIVA-SC in diverse contexts lies in understanding the specific roles and potential for health promotion that different social groups and CSOs may have in each setting (14).

## 5. Conclusions

PromoACTIVA-SC is the first validated tool developed to assess the role of civil society in health promotion. Through documentary analysis, expert review, cognitive interviews, and pilot testing, the tool identified eight phases and forty essential actions that reflect CSO engagement in the health promotion process. It offers both researchers and practitioners a structured framework to map practices, evaluate contributions, and identify areas for improvement.

Beyond measurement, the tool can be used by CSOs for self-assessment and capacity building, and by policymakers to strengthen collaboration with civil society in health promotion and disease prevention. By making CSO actions more visible and measurable, PromoACTIVA-SC can support accountability, encourage broader participation in health policy, and foster cross-sectoral approaches that contribute to more equitable and sustainable improvements in population health. Future applications of the tool across different settings will not only test its adaptability but also deepen understanding of how civil society can drive meaningful improvements in population health.

## Figures and Tables

**Table 1:** Types of CSOs activity (based on Greer et al., 2017) [14].

Activity	What	How
Policy	Engagement in decision-making and public policy	Representing interests Advocating for policies Pushing for the implementation of decisions Challenging other decisions Holding policymakers accountable (watchdog capacity that enhances public sector accountability)
Service	Provision of service directly to members and public	Identifying and addressing population needs
Governance	Explicit role in technical standard-setting; in professional and other self-regulation; and in corporatist arrangements for governing the economy	Formulating technology standards that enable the operation of key infrastructure and devices, or in the form of organizational practices. Measuring self-governance. Establishing social partnerships by assigning roles in societal organization, including governance in issues such as wage-setting, working conditions, and workforce training

**Table 2:** Cognitive interviews’ guide.

Strategies	Questions or Instructions
A. Thinking-aloud technique	Read each item aloud, explain its meaning, and articulate your understanding. What are your thoughts on the PromoACTIVA-SC tool extension? Do you find PromoACTIVA-SC clear and easily comprehensible?
B. Probing questions	Could you please explain the meaning of what you just read in your own words? Can you please rephrase the question in your own words? Could you please give me examples? Did you consider this question difficult to understand? Do you anticipate difficulty for others in your role in understanding this question? If the respondent answers hesitantly: - How confident are you in your response? - You said it depends… On what exactly?
C. Items requiring special attention: experts identified these as potentially challenging for respondents	Could you please describe in your own words what this item is asking? These were the items identified: Planning health promotion actions aimed at improving the health and/or training of citizens and civil society organizations. ‘Elaborating community health diagnosis to build healthy public policies and health promotion programs.’ If this is unclear, suggest the following wording: ‘Presenting proposals for the creation of health promotion policies and programs based on the analysis of indicators, needs, and strengths of the population’s health’. Advocating for the reorientation of the health sector. Advocating for funding and investment policies that support community associations.

**Table 3:** Characteristics of participants in the expert panel.

Participant	Expert Profession (Institution)	Theoretical Knowledge About Health Promotion Concepts	Belonging or Knowledge/Understanding of Civil Society
1 Male	Physician (Health Department, retired)	XXX	XXX
2 Female	Pharmacologist (University and CSOs)	XXX	XXX
3 Female	Nurse (manager and CSOs)	XXX	XXX
4 Female	Educator (University and CSOs)	XX	XXX
5 Female	Physician (Health department)	XXX	XXX
6 Female	Attorney (Local government and CSOs)	X	XXX
7 Male	Educator (CSOs)	XXX	XXX
8 Male	Psychologist (University and previously worked in CSOs)	X	XXX
9 Male	Sociologist (University)	XX	XXX
10 Male	Sociologist (Social Observatory Department)	X	XXX

The number of X indicates the level of expertise in the fields of health promotion, prevention or CSOs, with X denoting basic, XX proficient, and XXX advanced expertise.

**Table 4:** Items revision in content validation stage based on experts’ suggestions, after comprehensibility and semantic fit testing and pilot testing (**).

Items in Initial Version *	Items After Content Validity Analysis	Items After Comprehensibility and Semantic Fit Testing
*Items that experts proposed to unfold or simplify*		
3. Planning health promotion activities aimed at improving health promotion and/or empowering citizens, communities, and civil society organizations.	The decision was to maintain it, as it refers generally to the approach needed for planning health promotion actions.	
4. Collaborating in the identification of standards and competencies in the practice of health promotion.	The item was unfolded into two: - Collaborating to identify quality standards for health promotion. - Collaborating to identify training needs of health professionals for health promotion practice.	
19. Elaborating proposals for health promotion investment and monitoring budget execution.	The item was unfolded into two: - Elaborating proposals for investment in health promotion. - Monitoring budget execution in health promotion.	
20. Raising public awareness/opinion on healthy public policies among individuals and communities.	The item was simplified to: - Raising citizens’ awareness of healthy public policies.	
*Clarification of concepts (concepts in bold)*		
38. Developing and implementing actions to improve individuals’ **health literacy**.	- Developing and implementing actions to improve individuals’ knowledge and skills for health.	
36. Participating in the community health council		36 Participating in the community health council (citizen participation and representation body) 36. Participating in representation and citizen participation bodies in which aspects that influence health are dealt with (e.g., Health Councils, Commissions, and Forums) **
*Items needing more precision*		
1. Contributing to the design of the health strategy.	- Contributing to the design of the **local or regional** health strategy.	
2. Contributing to the planning of health promotion objectives.	- Contributing to the planning of the health promotion objectives within the Health System	
12. Developing shared leadership in health promotion.	- Developing shared leadership among organizational **members and other sectors** in health promotion.	
24. Advocating for changes in healthy public policies for the development of safe and healthy environments.	- Advocating for changes in healthy public policies to foster safe and healthy environments **for the community.**	
25. Advocating for the reorientation of the health sector.	- Advocating for the reorientation of the health sector **towards health promotion**.	
*Items that the experts suggest re-writing*		
7. Monitoring the implementation of health promotion activities and/or plan.	- Monitoring the implementation of **the planned** health promotion activities.	
9. Evaluating the internal organizational context.	- **Assessing internal organizational factors influencing health promotion practices**.	
16. Contributing to the development of health promotion know-how.	- **Sharing the practical knowledge for the implementation of health promotion**	
23. Acting as a watchdog of organizations and governments by publicizing their practices.	- **Conducting monitoring and control activities** on governments and organizational practices, **informing citizens about** these.	
*Minor changes for consistency in language*		
21. Raising awareness of community needs among policymakers and the scientific community	- Raising awareness of citizens’ health needs among policymakers and the scientific community.	
22. Mobilizing the community to advocate for healthy public policies.	- Mobilizing citizens to advocate for healthy public policies.	
*Items that experts suggest re-writing but no changes were made pending cognitive interviews*		
8. Elaborating on the **community health diagnosis** to build healthy public policies and health promotion programs.	Experts considered that the term in bold may not be clear to respondents.	8. Elaborating the community health diagnosis (Use and collection of sociodemographic, education, health, geographical, sociocultural problems, needs, and resources)’
26. Advocating for increasing **infrastructure** for health promotion.	Experts considered that the term in bold may not be clear to respondents.	Items 26 and 27 were merged. Participants considered the ‘infrastructure’ a ‘resource’. Final item: 27. Advocating for increasing resources for health promotion.
29. Advocating for funding and investment policies supporting community associations.	Experts considered that the item may not be clear to respondents.	
*Items eliminated*		
15. Developing coordination for health promotion.	It was included in items 33 and 34, which are more specific.	

* experts worked with the items in Spanish, which have been translated into English to facilitate reader comprehension (cultural translation not validated). ** item changed after the pilot testing.

**Table 5:** Characteristics of participants in the pilot testing stage.

Characteristics of the Organizations (N = 12)	
Years of existence of the organization	Mean 26.91 (SD = 11.09)
Size of the municipality they work on	
- <1000 inhabitants	16.7%
- Between 1000 and 5000 inhabitants	8.3%
- >5000 inhabitants	75%
Work also at regional level	83.3%
Work also at national or international level	58.3%
**Participants’ sociodemographic characteristics (N = 31)**	
Age (years-old)	43.13 (SD = 12)
Years affiliated with the organization	10.7 (SD = 10.33)
Monthly hours dedicated to the organization	122.03 (SD = 73.46)
Position in the organization:	
- Volunteer	16.1%
- Affiliate member	9.7%
- Paid employee	71%
- Other roles	3.2%
Role in the organization:	
- Coordination management or leadership	58.1%
- Direct interaction with groups/participants/patients…	29%
- Administrative duties	3.2%
- Other roles	9.7%
Level of education	
- High school/vocational training	19.4%
- University studies	80.6%
Work experience outside the organization	90.3%
Training in health promotion	74.2%

## Data Availability

Data available upon request from the authors.

## Supplementary Materials

The following supporting information can be downloaded at https://www.mdpi.com/article/10.3390/healthcare13172097/s1, Table S1: Search strategies used in the documentary analysis; Table S2: Codes after documentary analysis; Table S3: Categories and items resulting from the thematic framework analysis and refining stage. Table S4. Response percentages by items in the pilot testing stage; S1. Final version of PromoACTIVA-SCs1.

## Abbreviations

The following abbreviations are used in this manuscript:

CSOs	Civil Society Organizations
I-CVI	Content Validity Index for Item
NGO	Non-Governmental Organizations
SCVI/ave	Overall Content Validity Index
WHO	World Health Organization
