# Supplementary material for: PromoACTIVA-SC: A Tool Aiming at Identifying Health Promotion Practice of Civil Society Organizations
Source: Healthcare (Basel). 2025 Aug 23;13(17):2097. doi: 10.3390/healthcare13172097 (PMC12428060; doi:10.3390/healthcare13172097)
Supplement: Supplementary file 1 [file healthcare-13-02097-s001.zip › healthcare-3804023-supplementary.pdf]

healthcare

MDPI

PromoACTIVA-SC: A Tool Aiming at Identifying Health Promotion Practice of Civil Society Organizations

Table S1. Search strategies used in the documentary analysis.

<table>
<tr><th>Terms used in searches regarding Ottawa charter action areas</th><th>Terms related to health promotion</th><th>Action-related terms</th><th>Civil society terms</th><th>Number of documents found</th></tr>
<tr><td>Building healthy public Policy
'build* public healthy polic*' OR
'build* healthy public polic*' OR
'build healthy public policy' OR
'healthy public policy'</td><td rowspan="5">'health promotion'
OR
'health promotion and prevention'
OR
'positive health'</td><td rowspan="5">program* OR network* OR strateg* OR tool*

action* OR activity OR role

change* OR improv* OR develop*</td><td rowspan="5">Civil society organizations (CSOs)

Voluntary associations

Non-governmental organizations (NGOs)

Non-profit service providers

Foundations

Advocacy groups

Social movement organizations

Community groups

Self-help groups</td><td>57</td></tr>
<tr><td>Creating supportive environments
'supportive environment*' OR
'natural environment' OR
'built environment' OR
'psychosocial environment' OR
'economic environment' OR
'cultural environment'</td><td>16</td></tr>
<tr><td>Strengthening community action
'community empower*' OR
'community action*' OR
'community leadership' OR
'community decision' OR
'community health development' OR
'community health promotion' OR
'community capacity'</td><td>35</td></tr>
<tr><td>Developing personal skills
'skill* development' OR
'Developing personal skills' OR
'life skills'</td><td>163</td></tr>
<tr><td>Reorienting health services
reorient* OR transform* OR
restructur* OR reorienting health services
AND
health system OR
health care system OR health service* OR health care service*</td><td>38</td></tr>
</table>

Table S2. Codes after documentary analysis.

Ottawa Charter action areas	Codes identified in the documents
**BUILDING HEALTHY PUBLIC POLICY**	1. Agenda setting:<br>1.1. Lobbying efforts (advocacy)<br>1.2. Participates in direct negotiations with policymakers<br>1.3. Establishing norms for pressuring to prioritize policies<br>1.4. Helped to resolve tensions<br>1.5. Using evidence-based and rights-based approaches<br>1.6. Participating in Conferences<br>1.7. Using preexisting institutions to increase policymaker attention.<br>1.8. Assessing community health for public policies.<br>1.9. Establishing collaborations for cross-sectoral input into agenda setting<br>1.10. Community diagnosis<br>2. Generating public awareness/opinion of health promotion policies<br>3. Mobilizing community for advocacy for health promotion policies<br>4. Participation in policy formulation<br>5. Policy implementation:<br>5.1. Implementation<br>5.2. Monitoring for government accountability for the implementation<br>5.3. Employing strategies affecting government's reputation<br>6. Policy advocacy for funding/investment for community partnership<br>7. Participation in Policy Evaluation
**CREATING SUPPORTIVE ENVIRONMENTS**	8. Assessing characteristics of community environment<br>9. Advocacy for policy action: developing supportive environments<br>10. Advocacy for infrastructure changes (shared with STRENGTHENING COMMUNITY ACTION)<br>11. (Partnering in) designing community programs approaching environmental improvement<br>12. Building partnerships with other stakeholders to implement programs approaching environmental improvement.<br>13. Implementation of programs approaching environmental improvement.<br>14. Development of social networks<br>15. 'Dogwatching': supervision and rising awareness of organizational and government practices<br>16. Monitoring budgets<br>17. Rising awareness about the impact of the environment on health<br>-
**STRENGTHENING COMMUNITY ACTION**	18. Community Organization (goals, roles, activities)<br>19. Coalition Building<br>20. Building coalition capacity<br>21. Advocating for infrastructure changes (shared with CREATING SUPPORTIVE ENVIRONMENTS)<br>22. Engaging in forums and committees (to obtain resources) and voicing their views<br>23. Empowering residents, leaders, and the community at large<br>24. Advocacy training
**REORIENTING HEALTH SERVICES**	25. Extending (and partnering) attention towards health promotion<br>26. Advocacy for health sector reorientation<br>27. Increasing infrastructures for health promotion: developing partnerships and collaborating with stakeholders.

	28. Increasing health promotion infrastructures: broadening health care to promote health outside of the medical system.<br>29. Increasing health promotion infrastructures: coordinating for health promotion<br>30. Increasing health promotion infrastructures: to engage/mobilize neighborhoods/communities in change<br>31. Increasing resources for health promotion: grounded/experience/evidence/<br>32. Increasing resources for health promotion: innovation incubation<br>33. Increasing resources for health promotion: materials and tools<br>34. Building community capacity: coordinating for health promotion<br>35. Building community/health system capacity: health promotion training for increasing understanding/updating<br>36. Building community capacity: developing culture<br>37. Building community capacity: recruit volunteer leaders<br>38. Building civil society orgs capacity: shared leadership<br>39. Building organizational capacity: planning for health promotion<br>40. Planning for health promotion: collaborating on identifying standards (of care/coordination/professionals)<br>41. Planning for health promotion: contributing to service organization improvement
**DEVELOPING PERSONAL SKILLS**	42. Education, information, counseling<br>43. Developing health literacy actions (health and rights)<br>44. Developing health promotion programs (planning, implementation, and evaluation)<br>45. Partnering (with municipality) for improving individuals' referral to other services for skill development<br>46. Referring individuals to services (formal and informal)<br>47. Partnering for developing health promotion programs<br>48. Engaging local community in developing (planification, implementation, and evaluation) health promotion programs<br>49. Use asset mapping

Table S3. Categories and items resulting from the thematic framework analysis and refining stage.

Categories according to the type of activity	Final items	I-CVI Relevance
**PLANNING** Actions at the system and organizational levels aiming at formalizing, structuring, systematizing, and building capacity for the practice of health promotion, avoiding improvisation	1. Contributing to the design of the health strategy.	0.90
	2. Contributing to the planning of health promotion objectives.	1
	3. Planning health promotion activities aimed at improving health and/or empowering citizens, communities, and civil society organizations.	1
	4. Collaborating in the identification of standards and competencies for health promotion practice.	0.90
	5. Collaborating in the identification of coordination standards for health promotion development.	0.90
	6. Allocating budgetary resources within civil society organizations to fund health promotion.*	0.90
	7. Monitoring the implementation of health promotion activities and/or plans.*	1
**SITUATIONAL ANALYSIS** Actions aiming at characterizing and obtaining an in-depth knowledge of the initial situation at the individual, community and organizational levels to enable the formulation and planning of health promotion strategies.	8. Elaborating on the community health diagnosis to build healthy public policies and health promotion programs.	1
	9. Evaluating the internal organizational context.*	0.80
**DEVELOP ORGANIZATIONAL CAPACITY FOR HEALTH PROMOTION** Actions aiming at increasing and improving the organizational capacity for health promotion.	10. Contributing to the development of health promotion competencies among Health System professionals.	0.80
	11. Updating health promotion competencies of individuals within the organization.	0.90
	12. Developing shared leadership in health promotion.	0.80
	13. Building community capacity in health promotion.	1
	14. Identifying, recruiting, and empowering community leaders.	0.90
	15. Developing coordination for health promotion.	1
	16. Contributing to the development of health promotion know-how.	0.90
	17. Acting as an incubator for health promotion innovations.	0.80
	18. Developing materials and tools for health promotion practice.	0.90
	19.Elaborating proposals for health promotion investment and monitoring budget execution.	1

**GENERATE PUBLIC AWARENESS/OPINION FOR HEALTH PROMOTION.** Actions aiming at generating public awareness/opinion in individuals, community and system about people's rights and health determinants and their impact on health, for mobilization and advocacy for health promotion.	20.Raising public awareness/opinion on healthy public policies among individuals and communities.	0.90
	21.Raising awareness of community needs among policymakers and the scientific community.	1
	22.Mobilizing the community to advocate for healthy public policies.	0.90
	23.Acting as a watchdog of organizations and governments by publicizing their practices.	0.80
**ADVOCACY FOR HEALTH PROMOTION.** Actions aiming at advocating for the need for strategies, within and outside the health system, to promote health by addressing multiple determinants and respecting the rights of individuals and the principles of equity, participation and health in all policies.	24.Advocating for changes in healthy public policies for the development of safe and healthy environments.	1
	25.Advocating for the reorientation of the health sector.	0.40
	26.Advocating for increasing infrastructure for health promotion.	0.80
	27.Advocating for increasing resources for health promotion.	0.80
	28.Advocating for the prioritization of health promotion policies.	1
	29.Advocating for funding and investment policies supporting community association.	0.80
	30.Bringing health issues to the attention of policy makers.	0.80
**DEVELOPMENT OF NETWORKS FOR HEALTH PROMOTION** Actions aimed at developing both community and social networks. Social networks are understood as those relationships and links between individuals in a community that can facilitate access to or mobilization of social support for health. Community networks are those interactions between members of civil society and key agents that are developed under the same purpose and objectives, on a voluntary basis and with a range of local intervention in small territorial or population settings.	31.Developing social networks within the community.	1
	32.Referring individuals to available community services.	1

**DEVELOPMENT OF ALLIANCES, COALITIONS AND COLLABORATIONS FOR HEALTH PROMOTION** Actions aimed at identifying key actors for health promotion and building partnerships with them. Key stakeholders are citizens, health and social professionals, education or other key sectors, formal and informal associations of citizens, scientific and professional associations, the media, industry and local authorities.	33.Developing alliances, coalitions, and collaborations with key actors to shape public policies and healthy environments.	0.80
	34.Establishing alliances, coalitions and collaborations with key actors to implement health promotion programs.	0.90
	35.Establishing collaborations with researchers and universities.	1
	36.Participating in the community health council.*	1
**HEALTH PROMOTION INTERVENTION STRATEGIES** Actions aimed at improving the empowerment and/or health of individuals and communities.	37.Collaborating in formulating, implementing and evaluating healthy public policies	0.90
	38.Developing and implementing actions to improve individual's health literacy.	1
	39.Developing health promotion programs.	1
	40.Developing programs to promote individuals' positive interactions with the environment.	0.90

*Items added for completeness and relevance to national context and intersectoral collaboration.

Table S4. Response percentages by items in the pilot testing stage

	Items*	Response percentage according to scale values					
		1	2	3	4	5	**Percentage of Missing Data**
**Planning**	1. Contributing to the design of the local or regional health strategy.	22.6	16.1	19,4	16,1	22,6	3.2
	2. Contributing to the planning of health promotion objectives within the Health System.	22.6	25.8	12.9	19.4	16.1	3.2
	3. Planing health promotion activities aimed at improving health and/or empowering citizens, communities, and civil society organizations.	6.5	0	22.6	32.3	35.5	3.2
	4. Collaborating to identify quality standards for health promotion.	16.1	12.9	16.1	32.3	19.4	3.2
	5. Collaborating identifying training needs of health professionals for health promotion practice.	22.6	19.4	6,6	35.5	12.9	3.2
	6. Collaborating in the identification of coordination standards for health promotion development.	29	19.4	9.7	22.6	16.1	3.2
	7. Allocating budgetary resources within civil society organizations to fund health promotion.	22.6	9.7	19.4	25.8	19.4	3.2
	8. Monitoring the implementation of the planned health promotion activities.	16.1	9.7	9.7	32.3	29	3.2
**Situation analysis**	9. Elaborating the “Community Health Diagnosis” (Use and/or collection of sociodemographic, education, health, geographic, sociocultural data, problems, needs, and resources).	19.4	12.9	32.3	16.1	16.1	3.2
	10. Assessing internal organizational factors influencing health promotion practices.	12.9	12.9	35.5	19.4	16.1	3.2
**Develop organizational capacity**	11. Contributing to the development of health promotion competencies among Health System professionals.	16.1	16.1	9.7	32.3	25.8	0
	12. Updating health promotion competencies of individuals within the organization.	12.9	9.7	12.9	32.3	32.3	0
	13. Developing shared leadership among organizational members and other sectors in health promotion.	19.4	12.9	19.4	38.7	9.7	0
	14. Building community capacity in health promotion.	9.7	12.9	25.8	41.9	9.7	0
	15. Identifying, recruiting, and empowering community leaders.	19.4	16.1	19.4	29	16.1	0
	16. Sharing practical knowledge for the implementation of health promotion.	9.7	6.5	22.6	48.4	12.9	0
	17. Acting as an incubator for health promotion innovations.	12.9	3.2	29	38.7	16.1	0
	18. Developing materials and tools for health promotion practice.	3.2	9.7	9.7	**45.2**	**32.3**	0
	19. Elaborating proposals for investment in health promotion.	29	22.6	25.8	12.9	9.7	0
	20. Monitoring budget execution in health promotion.	32.3	12.9	16.1	19.4	19.4	0
**Ge ne rat**	21. Raising citizens’ awareness on healthy public policies.	6.5	6.5	38.7	32.3	16.1	0

	22. Raising awareness of citizens' health needs among policymakers and the scientific community.	12.9	12.9	22.6	32.3	19.4	0
	23. Mobilizing citizens to advocate for healthy public policies.	9.7	16.1	29	29	16.1	0
	24. Conducting monitoring and control activities on governments and organizational practices, informing citizens about these.	16.1	12.9	335.5	29	6.5	0
**Advocacy**	25. Advocating for changes in healthy public policies to foster safe and healthy environments for the community.	19.4	6.5	19.4	29	25.8	0
	26. Advocating for the reorientation of the health sector towards health promotion.	9.7	16.1	32.3	25.8	16.1	0
	27. Advocating for increasing resources for health promotion.	6.5	16.1	32.3	22.6	19.4	3.2
	28. Advocating for the prioritization of health promotion policies.	12.9	9.7	19.4	35.5	19.4	3.2
	29. Advocating for funding and investment policies that contribute to community association.	12.9	16.1	29	22.6	19.4	0
	30. Bringing health issues to the attention of policymakerss.	9.7	12.9	16.1	35.5	25.8	0
**Development of networks**	31. Developing social networks within the community.	16.1	3.2	12.9	51.6	16.1	0
	32. Referring individuals to available community services.	12.9	0	9.7	**25.8**	**51.6**	0
**Development of alliances, coalitions and collaborations**	33. Developing alliances, coalitions, and collaborations with key actors to shape public policies and healthy environments.	12.9	9.7	19.4	32.3	25.8	0
	34. Establishing alliances, coalitions and collaborations with key actors to implement health promotion programs.	6.5	16.1	19.4	32.3	25.8	0
	35. Establishing collaborations with researchers and universities.	16.1	12.9	19.4	29	22.6	0
	36. Participating in the community health council.	51.6	16.1	12.9	9.7	9.7	0
**Health promotion intervention strategies**	37. Collaborating in formulating, implementing, and evaluating healthy public policies	25.8	25.8	22.6	12.9	12.9	0
	38. Developing and implementing actions to improve individuals' knowledge and skills for health.	3.2	9.7	22.6	32.3	32.3	0
	39. Developing health promotion programs.	9.7	3.2	16.1	**41.9**	**29**	0
	40. Developing programs that encourage positive interaction between individuals and their environments.	3.2	3.2	35.5	22.6	35.5	0

* In PromoACTIVA, the items are written in Spanish, which have been translated into English to facilitate reader comprehension (cultural translation not validated).

S1. Final version of PromoACTIVA-SC

Cuestionario de Actividades en Promoción de la Salud en la Sociedad Civil (PromoACTIVA- SC)

Este cuestionario está dirigido a personas activistas, voluntarias, miembros o empleadas que participan en la gestión y/o lideran **organizaciones de la sociedad civil** con el objetivo de identificar las acciones de promoción de la salud que se llevan a cabo en las organizaciones. El cuestionario debe ser rellenado por una única persona.

En la primera parte del cuestionario se recogen datos sobre la organización y la persona que lo contesta con el fin de conocer a los participantes de este estudio. La segunda parte del cuestionario contiene ocho apartados que representan áreas de acción en promoción de salud que se ejecutan a través de 40 actividades redactadas en forma de ítems. En cada una de las 40 actividades tiene que señalar **el grado de ejecución por parte de la organización a la que pertenece de cada actividad propuesta**.

Si tiene cualquier problema, duda o comentario puede ponerse en contacto con el investigador responsable de este estudio por correo electrónico.

GRACIAS POR SU COLABORACIÓN

PRIMERA PARTE: Datos Socio-Demográficos

SOBRE LA ORGANIZACIÓN A LA QUE REPRESENTA

1. Nombre actual de la organización: ____________________
2. Página web de la organización (si la tiene): ____________________
3. Año de fundación o inicio de la organización (aaaa): ______
4. ¿Cómo describiría el objetivo principal de su organización?
   ____
   ____
5. La organización actúa en:
   - o Una localidad o barrio por debajo de 1000 habitantes
   - o Una localidad entre 1000 y 50.000 habitantes
   - o Una localidad con más de 50.000 habitantes
6. Además de a nivel local, ¿actúa su organización a nivel regional?:
   - o No
   - o Sí
7. ¿Su organización forma parte de alguna plataforma o federación nacional o internacional?
   - o No
   - o Sí

8. ¿Cómo describiría las principales actividades, programas y servicios que ofrece su organización? Por favor, elija las opciones que más se ajusten:
    - o Ofrecer información al público o a los grupos destinatarios
    - o Ofrecer ayuda práctica sobre temas concretos
    - o Ofrecer ayuda sobre aspectos de salud o enfermedad
    - o Organizar actividades sociales (de ocio o recreativas, culturales, artísticas)
    - o Otra descripción: ____

9. Cantidad aproximada de personas que forman parte de la organización:_______

10. ¿Recibe su organización algún tipo de financiación?
    - o No (pase a la pregunta 12)
    - o Sí

11. Indique la procedencia de dicha financiación:

	Si	No
Cuotas de afiliación o contribuciones de los miembros de la organización		
Ayudas o subvenciones del gobierno local o estatal		
Ayudas o subvenciones internacionales		
Ayudas a proyectos de entidades privadas		
Ingresos por eventos/campañas para recaudar fondos		
Donaciones		
Otros		

SOBRE USTED

12. Sexo:
    - o Hombre
    - o Mujer
    - o No binario

13. Año de nacimiento (aaaa): ______________________

14. Nivel de estudios:
    - o Educación Secundaria Obligatoria (ESO) o Graduado escolar
    - o Bachillerato/Formación profesional
    - o Estudios universitarios
    - o Otros: _____

15. ¿Qué rol ejerce en su organización? (marque solo la que considere que mejor le representa):
    - o Voluntario/a
    - o Persona afiliada a la organización
    - o Empleado/a remunerado/a
    - o Otro ______________________________

16. ¿Cuál es su tarea principal en la organización?
    - Coordinación, gestión o dirección de la organización
    - Trabajo directo con la población
    - Administración
    - Otros ________________________________

17. Año en el que comenzó a colaborar en organizaciones de la sociedad civil: (aaaa)_______

18. Año en el que comenzó a colaborar con esta organización: (aaaa)_______

19. Tiempo mensual de media que dedica a la organización: _______horas.

20. Si trabaja o ha trabajado fuera de la organización, indique en qué ámbito profesional:
    - Relacionado con la salud
    - Relacionado con lo social
    - Otras, indicar cual: ____________________________________
    - No aplicable

21. Ha tenido usted formación en promoción de salud:
    - No
    - Sí, indicar cual/es:____

SEGUNDA PARTE: Áreas de Acción y Actividades de Promoción de Salud

La segunda parte del cuestionario contiene ocho apartados que representan áreas de acción en promoción de salud que se ejecutan a través de 40 actividades redactadas en forma de ítems. En cada una de las 40 actividades tiene que señalar **su grado de ejecución por parte de la organización a la que pertenece**, rodeando la respuesta adecuada**.** El grado de ejecución de las actividades se valora a través de una escala de 5 puntos, siendo 1 el mínimo (ninguno) y 5 el máximo grado de ejecución (muy avanzado). Si usted no conoce si una actividad se lleva a cabo en su organización, por favor, señale la opción D "Desconozco". A continuación, se presenta una descripción aclaratoria de cada opción de respuesta. Por favor, léala atentamente:

1<br>Ninguno	2<br>Insuficiente	3<br>Suficiente	4<br>Avanzado	5<br>Muy avanzado	D<br>Desconozco
No se realiza dicha actividad.	Se está planteando la realización de la actividad.	La actividad está prevista y se ha iniciado parcialmente.	Se realiza la actividad. Su sistematización está pendiente.	La actividad se realiza de manera sistemática y está integrada en la práctica de la organización.	No sé si la actividad se lleva a cabo en nuestra organización.

Recuerde:

- Lea atentamente cada pregunta.
- Marque una única respuesta para cada pregunta, **rodeándola**.
- Conteste a todas las preguntas.
- No se trata de contestar sólo si usted mismo realiza esas actividades, si no de **si su organización las lleva a cabo**.

Área 1: PLANIFICACIÓN

Esta área incluye actividades por parte de su organización destinadas a formalizar, estructurar, sistematizar y crear capacidad para la práctica de la promoción de salud, evitando la improvisación.

	Ninguno	Insuficiente	Suficiente	Avanzado	Muy avanzado	Desconozco
1. Contribuir al diseño de la "Estrategia de Salud" local o regional (ej. Plan de Salud)	1	2	3	4	5	D
2. Contribuir a la planificación de objetivos de promoción de salud del Sistema de Salud	1	2	3	4	5	D
3. Planificar las actividades de promoción de salud de la propia organización	1	2	3	4	5	D
4. Colaborar con las autoridades competentes en la identificación de criterios de calidad en promoción de salud	1	2	3	4	5	D

5. Colaborar en la identificación de las necesidades formativas de los profesionales de la salud para la práctica de la promoción de salud	**1**	**2**	**3**	**4**	**5**	**D**
6. Colaborar en la identificación de criterios de coordinación para el desarrollo de la promoción de salud	**1**	**2**	**3**	**4**	**5**	**D**
7. Asignar una partida del presupuesto de la organización a la financiación de acciones de promoción de salud	**1**	**2**	**3**	**4**	**5**	**D**
8. Realizar el seguimiento de la implementación de las actividades de promoción de salud planificadas	**1**	**2**	**3**	**4**	**5**	**D**

Área 2: ANÁLISIS SITUACIONAL

Esta área incluye actividades de su organización destinadas a caracterizar y obtener un conocimiento profundo de la situación de partida a nivel individual, comunitario y organizacional que permita formular y planificar estrategias de promoción de salud.

	**Ninguno**	**Isuficiente**	**Suficiente**	**Avanzado**	**Muy avanzado**	**Desconozco**
9. Realizar el "Diagnóstico de Salud Comunitaria" (Uso y/o recogida de datos sociodemográficos, de educación, salud, geográficos, socioculturales, problemas, necesidades y recursos).	**1**	**2**	**3**	**4**	**5**	**D**
10. Evaluar los factores de la propia organización que influyen en la práctica de la promoción de salud	**1**	**2**	**3**	**4**	**5**	**D**

Área 3: DESARROLLO DE CAPACIDAD ORGANIZACIONAL PARA LA PROMOCIÓN DE LA SALUD

Esta área incluye actividades de su organización destinadas a incrementar y mejorar la distribución y coordinación de los recursos humanos, relacionales, materiales, estructurales y financieros, requeridos para la realización de actividades de promoción de salud

	Ninguno	Insuficiente	Suficiente	Avanzado	Muy avanzado	Desconozco
11. Contribuir a la formación en promoción de salud de los profesionales del Sistema Sanitario	**1**	**2**	**3**	**4**	**5**	**D**
12. Actualizar la formación en promoción de salud de los miembros de la organización.	**1**	**2**	**3**	**4**	**5**	**D**
13. Desarrollar el liderazgo compartido con los distintos miembros de la organización y con otros sectores en promoción de salud	**1**	**2**	**3**	**4**	**5**	**D**
14. Crear capacidad en la comunidad para la promoción de salud	**1**	**2**	**3**	**4**	**5**	**D**
15. Identificar, reclutar y empoderar líderes comunitarios	**1**	**2**	**3**	**4**	**5**	**D**
16. Compartir la experiencia de la organización para facilitar la implementación de la promoción de salud	**1**	**2**	**3**	**4**	**5**	**D**
17. Actuar como organización que da forma y desarrolla proyectos innovadores en promoción de salud	**1**	**2**	**3**	**4**	**5**	**D**
18. Elaborar materiales y herramientas para la práctica de promoción de salud	**1**	**2**	**3**	**4**	**5**	**D**
19. Elaborar propuestas de inversión en promoción de salud	**1**	**2**	**3**	**4**	**5**	**D**
20. Realizar el seguimiento de la ejecución de los presupuestos	**1**	**2**	**3**	**4**	**5**	**D**

Área 4: GENERAR CONCIENCIA PARA LA PROMOCIÓN DE LA SALUD

Esta área incluye actividades de su organización destinadas a generar conciencia en la ciudadanía y el sistema sobre los derechos de las personas y los determinantes de salud y su repercusión en la misma, para su movilización y abogacía para la promoción de salud.

	Ninguno	Insuficiente	Suficiente	Avanzado	Muy avanzado	Desconozco
21. Concienciar a la ciudadanía sobre el impacto de las políticas públicas saludables	**1**	**2**	**3**	**4**	**5**	**D**
22. Concienciar a las autoridades y a la comunidad científica sobre las necesidades de la ciudadanía	**1**	**2**	**3**	**4**	**5**	**D**
23. Movilizar a la ciudadanía para que abogue por políticas públicas saludables	**1**	**2**	**3**	**4**	**5**	**D**
24. Realizar actividades de seguimiento y control de los gobiernos y las organizaciones dando a conocer a la ciudadanía las prácticas de estos	**1**	**2**	**3**	**4**	**5**	**D**

Área 5: ABOGACÍA PARA LA PROMOCIÓN DE LA SALUD

Esta área incluye actividades de su organización dirigidas a defender la necesidad de estrategias, dentro y fuera del sistema de salud, que permitan promover la salud atendiendo a los múltiples determinantes y respetando los derechos de las personas y los principios de equidad, participación y salud en todas las políticas.

	Ninguno	Insuficiente	Suficiente	Avanzado	Muy avanzado	Desconozco
25. Abogar por cambios en las políticas públicas saludables para el desarrollo de entornos seguros y saludables para la comunidad	**1**	**2**	**3**	**4**	**5**	**D**
26. Abogar por la reorientación del sector salud hacia la promoción de salud	**1**	**2**	**3**	**4**	**5**	**D**
27. Abogar por el incremento de recursos para la promoción de salud	**1**	**2**	**3**	**4**	**5**	**D**

28. Abogar por la priorización de políticas de promoción de salud	**1**	**2**	**3**	**4**	**5**	**D**
29. Abogar por políticas de financiación e inversión que contribuyan al asociacionismo de la comunidad	**1**	**2**	**3**	**4**	**5**	**D**
30. Captar la atención de los políticos sobre temas de salud	**1**	**2**	**3**	**4**	**5**	**D**

Área 6: DESARROLLO DE REDES PARA LA PROMOCIÓN DE SALUD

Esta área incluye actividades de su organización dirigidas al desarrollo de redes sociales. Se entiende por redes sociales aquellas relaciones y vínculos entre los individuos de una comunidad que pueden facilitar el acceso o movilización del soporte social a favor de la salud.

	**Ninguno**	**Insuficiente**	**Suficiente**	**Avanzado**	**Muy avanzado**	**Desconozco**
31. Desarrollar redes sociales que potencien la salud en la comunidad	**1**	**2**	**3**	**4**	**5**	**D**
32. Derivar a las personas a los servicios disponibles en la comunidad	**1**	**2**	**3**	**4**	**5**	**D**

Área 7: DESARROLLO DE ALIANZAS, COALICIONES Y COLABORACIONES PARA LA PROMOCIÓN DE LA SALUD

Esta área incluye actividades de su organización dirigidas a identificar los agentes clave para la promoción de salud y construir colaboraciones con los mismos. Son agentes clave la ciudadanía, los/las profesionales de la salud, de la educación u otros sectores clave, las asociaciones formales e informales de personas, asociaciones científicas y profesionales, los medios de comunicación, la industria y las autoridades locales.

	**Ninguno**	**Insuficiente**	**Suficiente**	**Avanzado**	**Muy avanzado**	**Desconozco**
33. Desarrollar alianzas, coaliciones y colaboraciones con otros agentes clave para la construcción de políticas públicas y entornos saludables	**1**	**2**	**3**	**4**	**5**	**D**
34. Establecer alianzas, coaliciones y colaboraciones con otros agentes clave para la implementación de programas de promoción de salud	**1**	**2**	**3**	**4**	**5**	**D**

35. Establecer colaboraciones con investigadores/as y universidades	**1**	**2**	**3**	**4**	**5**	**D**
36. Participar en órganos de representación y participación ciudadana en los que se trabajen aspectos que influyen en la salud (Ej. Consejos, Comisiones y Foros de Salud)	**1**	**2**	**3**	**4**	**5**	**D**

Área 8: ESTRATEGIAS DE INTERVENCIÓN EN PROMOCIÓN DE LA SALUD

Esta área incluye actividades de su organización destinadas a mejorar la capacitación y/o la salud de la ciudadanía y comunidades.

	**Ninguno**	**Insuficiente**	**Suficiente**	**Avanzado**	**Muy avanzado**	**Desconozco**
37. Colaborar en la formulación, implementación y evaluación de políticas públicas saludables	**1**	**2**	**3**	**4**	**5**	**D**
38. Desarrollar e implementar acciones que mejoren el nivel de conocimientos y habilidades para la salud de las personas	**1**	**2**	**3**	**4**	**5**	**D**
39. Desarrollar programas de promoción de salud	**1**	**2**	**3**	**4**	**5**	**D**
40. Desarrollar programas que promuevan una interacción positiva de las personas con el entorno (físico, social, ambiental, cultural, etc.)	**1**	**2**	**3**	**4**	**5**	**D**
